# Mapping the intellectual structure of the refrigerated vehicle routing problem: research perspectives and structural knowledge gaps

**DOI:** 10.3389/frma.2026.1817900

**Published:** 2026-05-29

**Authors:** Daniel Aristizábal Torres, César Augusto Peñuela Meneses, Jhon Jairo Santa Chávez, Luis Miguel Escobar Falcón

**Affiliations:** 1 Faculty of Engineering, Universidad Libre Seccional Pereira, Pereira, Colombia; 2 Doctoral Program in Engineering, Universidad Tecnológica de Pereira, Pereira, Colombia; 3 Physical Engineering, Universidad Tecnológica de Pereira, Pereira, Colombia

**Keywords:** co-citation network analysis, cold chain logistics, cold chain optimization, refrigerated vehicle routing problem, research gaps, sustainable routing

## Abstract

**Introduction:**

The Refrigerated Vehicle Routing Problem (RVRP) has evolved from a specialized extension of the classical Vehicle Routing Problem into a multidisciplinary research domain integrating cold chain logistics, sustainability, inventory coordination, and dynamic intelligent systems. Despite its rapid growth, the field's intellectual structure remains fragmented across methodological traditions, limiting cross-perspective integration.

**Methods:**

This study maps the evolution of the RVRP through a co-citation network analysis of 469 articles indexed in the Web of Science Core Collection. Bibliographic data were processed using the R-TOS (Tree of Science for R) package and analyzed in Gephi, generating a network comprising 3,116 nodes and 11,341 directed edges. Modularity-based community detection, PageRank centrality analysis, and keyword frequency examination were employed to identify dominant research streams and structural patterns.

**Results:**

The results reveal a cohesive yet moderately modular intellectual architecture, from which four dominant research perspectives emerge: (1) algorithmic optimization and mathematical modeling, (2) perishable logistics and cold chain operational management, (3) inventory–routing integration and strategic coordination, and (4) dynamic, intelligent, and multi-objective routing systems. Cross-perspective synthesis highlights increasing methodological sophistication but persistent fragmentation, particularly in the limited integration of thermodynamic variability, deterioration dynamics, and energy consumption modeling.

**Conclusion:**

A critical structural gap identified across perspectives is the widespread assumption of constant temperature along routing arcs, which constrains the physical realism of many optimization models. The findings suggest that future RVRP research should move toward systemic integration, coupling routing decisions with dynamic thermal modeling, environmental variability, and energy–thermodynamic interactions. By providing a reproducible scientometric framework and a structured synthesis of emerging knowledge gaps, this study contributes to the theoretical consolidation and future development of refrigerated routing research.

## 1 Introduction

The Vehicle Routing Problem (VRP) constitutes one of the foundational problems in operations research, originating from the seminal truck dispatching formulation of Dantzig and Ramser (1959). Since then, the VRP has evolved into a broad family of increasingly complex variants incorporating time windows, stochastic demand, and multi-objective optimization frameworks (Golden et al., 2008; Toth and Vigo, 2014). In its classical structure, the VRP involves decisions over a set of binary routing variables that determine which vehicle traverses each arc of a distribution network, subject to vehicle capacity constraints, route continuity conditions, and cost minimization objectives.

Within this expanding methodological landscape, the Refrigerated Vehicle Routing Problem (RVRP) has emerged as a specialized extension driven by the operational demands of cold chain logistics and perishable goods distribution. The RVRP extends the classical VRP structure by introducing additional decision dimensions and constraints absent from standard formulations: temperature-dependent product deterioration functions that link delivery sequencing to quality loss, refrigeration energy consumption terms that couple routing decisions with environmental performance, and cold chain feasibility constraints that enforce thermal compliance throughout the distribution arc (Escobar et al., 2022; Meneghetti and Ceschia, 2020). These extensions reposition the RVRP as a fundamentally multidisciplinary problem spanning combinatorial optimization, food engineering, and environmental logistics, a breadth that is structurally captured by the four research perspectives identified in this study.

Unlike classical VRP formulations, the RVRP must simultaneously ensure routing efficiency, temperature control, product quality preservation, and energy performance. This dual operational–thermodynamic nature significantly increases modeling complexity. Recent studies have proposed advanced metaheuristic approaches specifically tailored to refrigerated logistics, demonstrating how problem-specific neighborhood structures and search strategies can effectively address the combinatorial complexity of homogeneous fleet configurations (Escobar et al., 2022). In parallel, energy-efficient formulations have explicitly incorporated refrigeration energy consumption into routing decisions, thereby linking transport optimization with environmental performance and sustainability objectives (Meneghetti and Ceschia, 2020). These contributions illustrate the dual evolution of the field toward both algorithmic refinement and sustainability integration within refrigerated transport systems.

The cold chain refers to the temperature-controlled supply chain infrastructure required to maintain the quality and safety of perishable products from production to final consumption (Hsiao et al., 2018; James et al., 2006). It encompasses refrigerated storage facilities, transportation systems with active or passive cooling mechanisms, temperature monitoring technologies, and regulatory compliance processes (Hsu et al., 2007; Wang et al., 2018). Operational challenges in cold chain management include maintaining consistent temperature ranges across heterogeneous geographic and climatic conditions, minimizing energy consumption during refrigeration, and managing product deterioration as a function of thermal exposure time (Govindan et al., 2014; Rong et al., 2011). These challenges are compounded in the RVRP context, where routing decisions directly interact with thermodynamic constraints, product shelf life, and energy performance, making the RVRP a fundamentally multi-disciplinary problem at the intersection of operations research, food engineering, and environmental logistics (Escobar et al., 2022; Meneghetti and Ceschia, 2020).

Beyond algorithmic advancements, sustainability-oriented RVRP models have incorporated carbon emissions and environmental objectives into routing formulations. For instance, multi-objective frameworks integrating environmental costs and logistics performance have been developed to capture trade-offs between operational efficiency and emissions reduction (Guo et al., 2022; Liu C. et al., 2020; Wang et al., 2017). These studies extend traditional cost-minimization paradigms toward environmentally aware routing strategies.

Simultaneously, research has advanced the integration of routing decisions with inventory planning and multi-period coordination mechanisms. Inventory–routing formulations have expanded the problem scope by embedding transport within broader supply chain decision systems (Raa and Aghezzaf, 2009; Soysal et al., 2015; Yu and Nagurney, 2013). These approaches reposition routing as a component of systemic logistics coordination rather than an isolated combinatorial problem.

Dynamic and time-dependent routing models have further enriched the RVRP literature by incorporating traffic variability and adaptive decision-making. Time-dependent and dynamic routing formulations have been explored in transportation research to better reflect real-world operational environments (Erdogan and Miller-Hooks, 2012; Liu et al., 2013; Xiao and Konak, 2016). Moreover, advanced metaheuristics and hybrid computational intelligence approaches have been proposed to address large-scale and complex variants of the problem (Liu G. et al., 2020; Song et al., 2020; Zhang H. et al., 2019).

Despite this substantial body of work, the intellectual structure of the RVRP domain remains fragmented across methodological traditions. Algorithmic optimization, cold chain deterioration modeling, sustainability-oriented extensions, and systemic integration approaches have progressed in parallel, yet their structural interconnections have not been systematically examined. Existing reviews tend to classify RVRP variants thematically, but they rarely provide a formal mapping of the citation architecture that underpins the field's development.

To address this limitation, the present study conducts a co-citation network analysis of RVRP research indexed in the Web of Science Core Collection. By combining modularity-based community detection, PageRank centrality filtering, and keyword frequency analysis, this article identifies four dominant research perspectives shaping the evolution of the RVRP and highlights emerging structural research gaps, particularly the persistent assumption of constant temperature along routing arcs and the limited integration between thermodynamic variability, deterioration dynamics, and energy consumption modeling. Through this data-driven structural synthesis, the study contributes to the theoretical consolidation of refrigerated routing research and lays the foundation for more integrated, physically grounded modeling frameworks.

It is important to acknowledge the epistemological positioning of co-citation network analysis relative to traditional narrative review approaches. Co-citation analysis does not replace critical reading of the primary literature; rather, it operates at a structural level that narrative reviews cannot systematically access. While narrative reviews synthesize content through the researcher's interpretive selection of individual works, co-citation analysis maps the citation architecture collectively constructed by the research community through thousands of independent publication decisions—revealing structural patterns, intellectual communities, and knowledge gaps that are not visible from the reading of individual articles. At the same time, this method has recognized limitations: it depends on the coverage of the source database, it captures structural proximity rather than semantic similarity, and its outputs require sustained domain-informed interpretation to yield meaningful conclusions. In this study, the identification of the four research perspectives, their internal logic, and their associated knowledge gaps reflects the combined use of structural evidence and critical engagement with the primary literature. The goal is not to substitute narrative synthesis but to provide a reproducible, data-driven structural complement to it.

Rather than adopting a conventional narrative literature review, this study employs co-citation network analysis as a methodologically rigorous and reproducible alternative (Small, 1973; Aria and Cuccurullo, 2017; Barrera Rodríguez et al., 2023). This approach enables a data-driven structural synthesis of the field, revealing the citation architecture and intellectual interdependencies that underpin the RVRP's evolution, a contribution that thematic narrative reviews are structurally limited to provide (Zupic and Cater, 2015; Donthu et al., 2021a).

## 2 Materials and methods

### 2.1 Data source and search strategy

**Figure 1:**
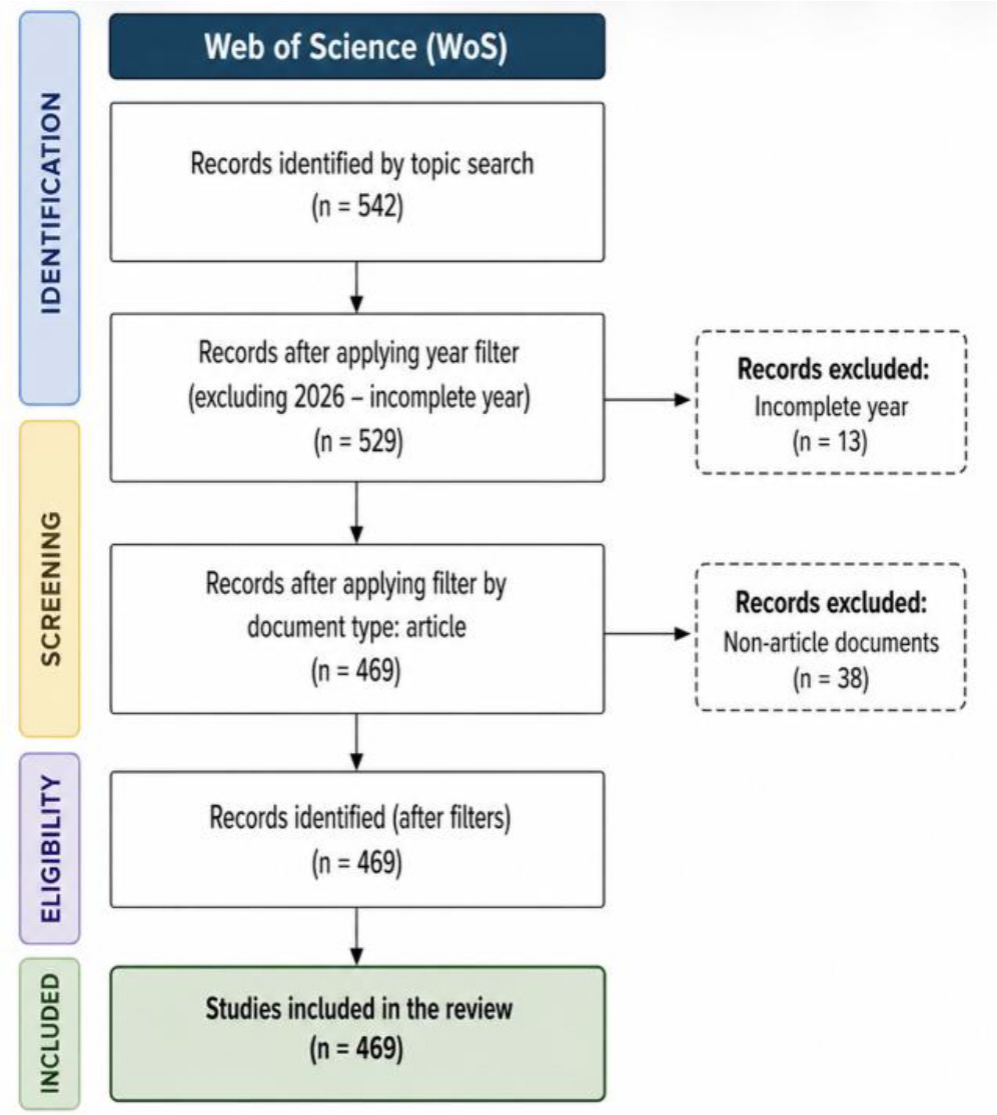
PRISMA-inspired flow diagram of the literature search and screening process.

The bibliographic dataset was retrieved from the Web of Science Core Collection (WoS) database due to its high indexing standards and citation reliability. The search was conducted on February 11, 2026, using the following query restricted to peer-reviewed journal articles: (“vehicle routing problem” OR VRP) AND (refrig^*^ OR cold OR fresh OR perishable). The initial search retrieved 542 records. Subsequently, two sequential filters were applied following the PRISMA (Preferred Reporting Items for Systematic Reviews and Meta-Analyses) framework: (i) an year filter excluding records from 2026 as an incomplete publication year, removing 13 records and yielding 529 publications; and (ii) a document-type filter retaining only journal articles, removing 38 records of other document types, resulting in a final dataset of 469 publications. The complete search and screening process is documented in the PRISMA flow diagram presented in Figure 1. Full records and cited references were exported in plain text (.txt) format, including complete bibliographic information and references, to enable co-citation network construction.

### 2.2 Data processing and network construction

R-TOS (package available via CRAN/GitHub) was selected over alternative bibliometric tools, including bibliometrix, VOSviewer, and CiteSpace, for its capacity to directly process Web of Science plain-text exports into weighted, directed co-citation networks exportable to Gephi (Gephi Consortium; https://gephi.org) in GraphML format, preserving full citation directionality. Unlike VOSviewer, which performs its own internal layout and clustering, R-TOS decouples network construction from visualization and analysis, enabling greater methodological transparency and flexibility in applying community detection algorithms. This methodological pipeline has been successfully applied in bibliometric structural analyses across adjacent research domains (Barrera Rodríguez et al., 2023; Robledo et al., 2023), validating its appropriateness for mapping the intellectual structure of emerging interdisciplinary fields such as the RVRP.

The exported Web of Science file was processed using the R-TOS (Tree of Science for R) package. The tool was employed to convert the raw WoS bibliographic data into a structured network format compatible with Gephi. Specifically, the dataset was transformed into a GraphML file representing the co-citation relationships among cited references.

The resulting graph represents cited references as nodes and co-citation relationships as directed edges, forming the basis for subsequent network analysis.

### 2.3 Network analysis and visualization

The generated GraphML file was imported into Gephi for network visualization and structural analysis. The network comprises 3,116 nodes and 11,341 directed edges.

To spatially distribute nodes according to structural proximity, the Force Atlas 2 layout algorithm was applied. This force-directed layout enhances the visualization of densely connected regions and facilitates the identification of structural communities.

To characterize the network's global topology, structural metrics, including network diameter and average degree, were computed. These measures provide insights into network cohesion, connectivity, and intellectual dispersion.

### 2.4 Community detection

Community detection was performed using modularity optimization in Gephi. The modularity algorithm partitions the network into clusters by maximizing intra-cluster density while minimizing inter-cluster connections. This procedure enabled the identification of structurally cohesive sub-networks within the co-citation structure. Four dominant communities were detected, representing differentiated research streams within the RVRP domain.

### 2.5 Perspective identification and interpretative framework

To strengthen the interpretative robustness of each detected community, the PageRank centrality measure was computed. PageRank was selected because it captures not only the number of citation links but also the structural influence of each reference within the network. For each detected cluster, the references with the highest PageRank values were examined in detail to identify the intellectual core of the respective research perspective.

To complement the structural interpretation, lexical analysis was conducted using the R-TOS tool. Word clouds were constructed for each community based on the frequency of author keywords associated with the references belonging to each cluster. The keyword frequency distribution enabled the identification of dominant thematic patterns within each perspective.

This combined approach, based on modularity community detection, PageRank centrality filtering, and keyword frequency analysis, provided a structured and reproducible framework for defining the four research perspectives.

It is important to note that no additional exclusion criteria were applied beyond the initial data filtering described in Section 2.1. All references processed in the co-citation network were retained for structural analysis; the focus on four dominant perspectives reflects the structural hierarchy of the network rather than any *post-hoc* exclusion of data.

## 3 Results

### 3.1 Temporal evolution of RVRP publications

**Figure 2:**
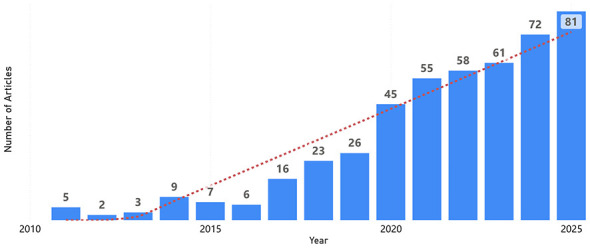
Temporal evolution of RVRP publications in web of science.

Figure 2 presents the temporal evolution of publications related to the Refrigerated Vehicle Routing Problem (RVRP) indexed in the Web of Science Core Collection according to the search equation defined in Section 1. The dataset reveals a sustained and accelerated growth trajectory over the last decade.

During the early years of the observation period, the number of annual publications remained limited, reflecting the specialized and emerging nature of refrigerated routing research. However, beginning around 2018–2019, a marked increase in scholarly output becomes evident. This acceleration coincides with the growing integration of sustainability concerns, carbon-aware logistics, digital cold chain monitoring technologies, and advanced multi-objective optimization frameworks.

The exponential growth pattern observed in recent years suggests that the RVRP has transitioned from a niche extension of the classical Vehicle Routing Problem to a consolidated and expanding research domain. This rapid expansion justifies the need for a structural scientometric analysis capable of identifying dominant research streams, intellectual foundations, and emerging knowledge gaps within the field.

Table 1 presents the ten most productive authors in the RVRP literature within the analyzed corpus, along with their institutional affiliations, total citations, H-index, and year of first publication. The results reveal a strong geographic concentration, with six of the ten most productive authors affiliated with Chinese institutions, particularly Chongqing University. Portugal and Iran each contribute one author among the top ten, reflecting a predominantly Asian and European authorship structure. Notably, Diabat (561 citations) and Almada-Lobo (466 citations) lead in terms of citation impact, suggesting that their contributions on perishable logistics and inventory-routing integration have exerted significant structural influence on the field's development.

**Table 1:** Most productive authors in RVRP research (web of science core collection, February 11, 2026).

Rank	Author	Number of publications	Total citations	H-index	First year	Affiliation
1	Li, Y.	9	346	8	2015	Chongqing University (China)
2	Almada-Lobo, B.	6	466	6	2013	Universidade do Porto (Portugal)
3	Diabat, A.	6	561	6	2013	Masdar Institute of Science and Technology (United Arab Emirates)
4	Lim, M.K.	6	343	6	2019	Chongqing University (China)
5	Wu, D.Q.	6	131	6	2021	Shanghai Ocean University (China)
6	Tavakkoli-Moghaddam, R.	6	151	5	2017	University of Tehran (Iran)
7	Zhang, J.	6	254	5	2014	Chongqing Jiaotong University (China)
8	Zhang, Y.	8	176	5	2014	Tsinghua University (China)
9	Amorim, P.	5	388	5	2013	Institute for Systems and Computer Engineering, Technology and Science (Portugal)
10	Leng, L.L.	5	160	5	2020	Hangzhou City University (China)

### 3.2 Network structure

**Figure 3:**
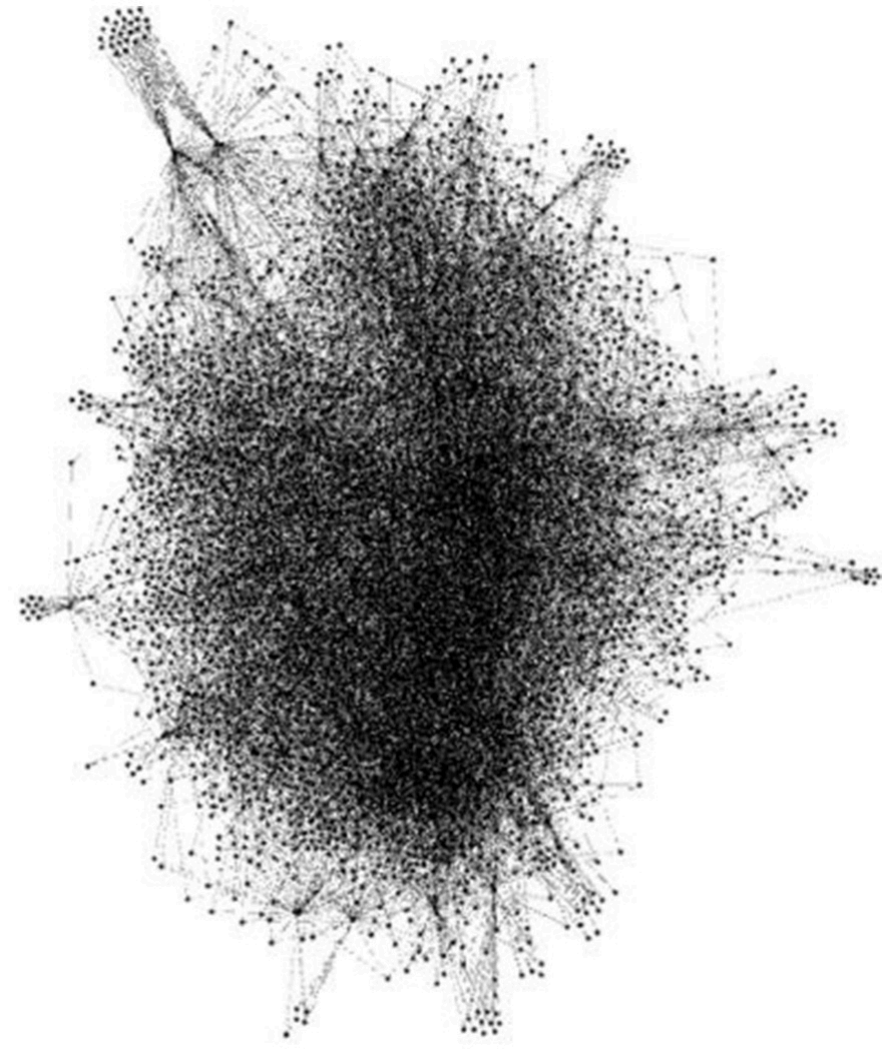
Global structure of the RVRP co-citation network (unclustered visualization).

The co-citation network constructed from the selected RVRP literature comprises 3,116 nodes and 11,341 directed edges, representing cited references and their co-citation relationships. The network was developed and analyzed in Gephi, using the Force Atlas 2 layout algorithm to spatially distribute nodes according to their structural proximity. Figure 3 presents a global, unclustered visualization of the network, highlighting its core–periphery structure and the dense concentration of highly interconnected references at its center.

Figure 2 serves a dual purpose in the structural analysis. First, it confirms the validity of the dataset as a cohesive citation network suitable for community detection, evidenced by the clearly defined core–periphery configuration emerging from the Force Atlas 2 layout. Second, it visually anticipates the intellectual architecture characterized analytically in subsequent sections: the dense central region corresponds to highly co-cited foundational references that exert structural influence across multiple research streams, while peripheral nodes represent more specialized or recently incorporated contributions with limited cross-cluster co-citation links. Together, these features establish the network as both structurally compact and thematically diverse, justifying the subsequent application of modularity-based community detection.

To characterize the network's global topology, several structural metrics were computed, including the network diameter and average degree. The network diameter indicates the longest geodesic distance between two nodes, reflecting the breadth of the intellectual structure, while the average degree measures the overall connectivity among references. These indicators reveal a highly interconnected and cohesive citation architecture with a dense central core and peripheral extensions.

The structural metrics reported in Table 2 indicate that the RVRP co-citation network exhibits a cohesive yet moderately modular architecture. The low density (0.0023) and average degree (3.64) are consistent with large-scale academic citation networks, where connections are sparse relative to the total number of possible links. However, the average path length (2.82) and network diameter (8) suggest that the intellectual structure remains compact, with relatively short geodesic distances between references. This combination reflects a dense core–periphery configuration in which foundational works remain structurally proximate across thematic boundaries.

**Table 2:** Structural properties of the RVRP co-citation network.

Metric	Value	Interpretation in the RVRP context
Number of nodes	3,116	Each node represents a cited reference; the large size confirms broad intellectual scope
Number of edges	11,341	Co-citation links between references; high count reflects active cross-referencing across studies
Network density	0.0023	Sparse connectivity consistent with large citation networks; indicates no single paradigm dominates the field
Average degree	3.64	Each reference is co-cited with approximately 4 others on average, reflecting moderate cross-fertilization
Network diameter	8	Maximum intellectual distance between any two references; value of 8 in 3,116 nodes confirms a compact structure
Average path length	2.82	References are reachable within 3 citation steps, confirming a cohesive intellectual core
Modularity (*Q*)	0.474	Values above 0.4 indicate well-defined community structure; confirms meaningful subfields exist within the RVRP domain
Number of communities	9	Four dominant and five peripherals; hierarchy reflects structural centrality differences among research streams
Largest community	551 nodes (17.7%)	Confirms no single perspective monopolizes the field; intellectual structure is distributed across multiple streams

The modularity value (*Q* = 0.474) indicates a well-defined community structure, confirming the presence of meaningful subfields within the broader RVRP domain. Although the modularity algorithm identified nine communities, closer inspection reveals that only four clusters concentrate the majority of structurally central and highly cited references. The remaining five communities correspond to smaller, more specialized or peripheral subthemes with limited cross-cluster influence. Consequently, for interpretative clarity and theoretical consolidation, the analysis focuses on the four dominant communities that represent the principal research perspectives shaping the field's evolution. This aggregation does not ignore the existence of additional subclusters but rather reflects a structural hierarchy in which major intellectual streams coexist with secondary, emerging, or niche research directions.

The scale of the resulting network (3,116 nodes; 11,341 edges) is consistent with co-citation analyses of specialized subfields within logistics and operations research, where corpus-dependent network size reflects domain scope rather than a universal benchmark (Zupic and Cater, 2015; Donthu et al., 2021b). The modularity value (*Q* = 0.474) and average path length (2.82) are within the ranges conventionally associated with well-defined community structure and small-world topology in academic co-citation networks (Aria and Cuccurullo, 2017).

Community detection was subsequently performed using modularity optimization, thereby identifying structurally cohesive clusters within the network. The modularity-based partition revealed four dominant communities, which were interpreted as distinct research perspectives within the RVRP domain. These clusters are analyzed in the following section.

### 3.3 Perspective identification

**Figure 4:**
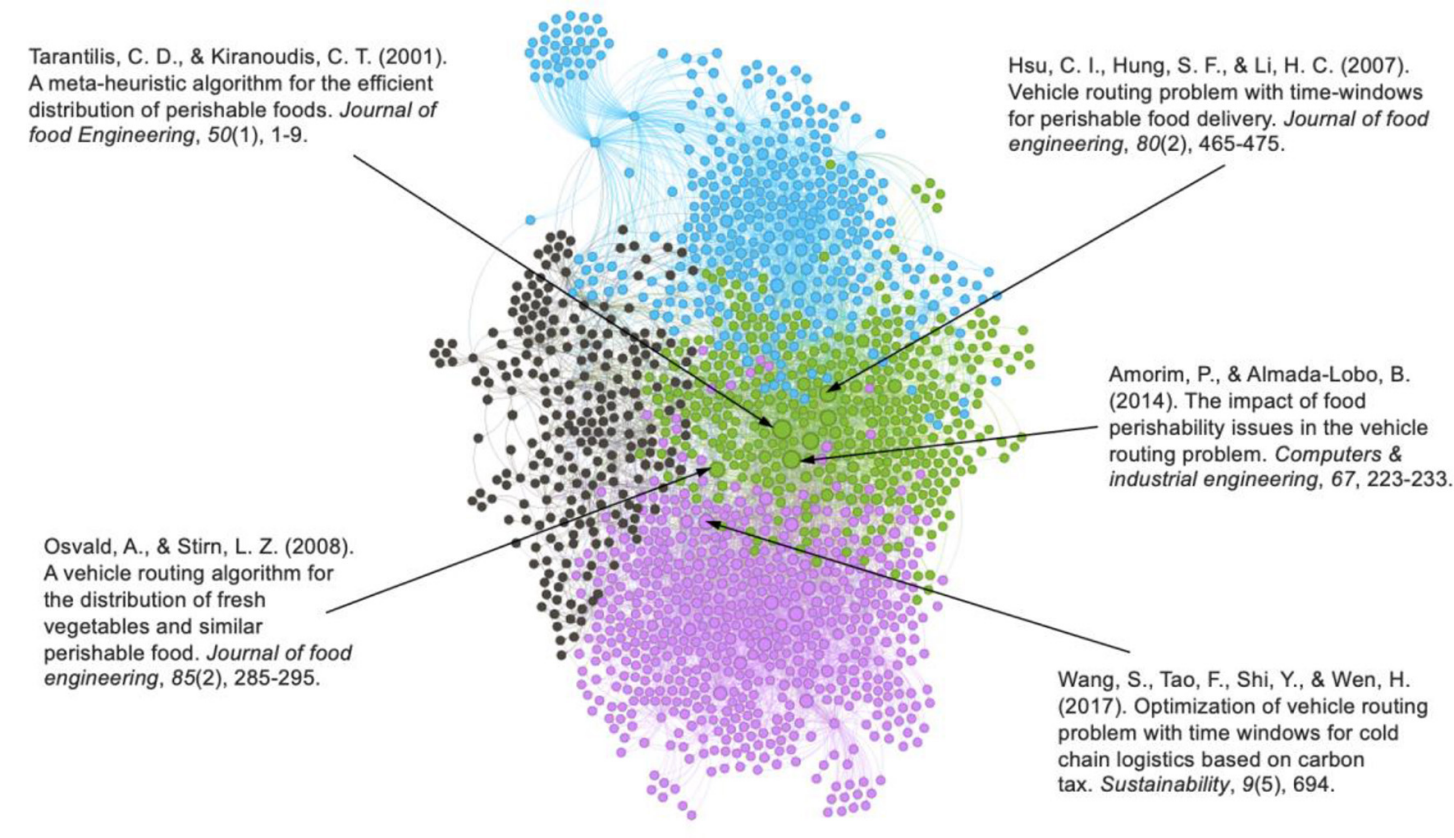
Co-citation network of the RVRP literature displaying the main research perspectives and their most influential references.

Community detection applied to the co-citation network revealed four structurally distinct research perspectives within the RVRP literature, as illustrated in Figure 4. Each cluster exhibits high intra-community co-citation density and low inter-community connectivity, confirming thematic cohesion across research streams. The four main perspectives are analyzed in detail in the following subsections.

#### 3.3.1 Perspective 1. Algorithmic optimization and mathematical modeling of the RVRP

**Figure 5:**
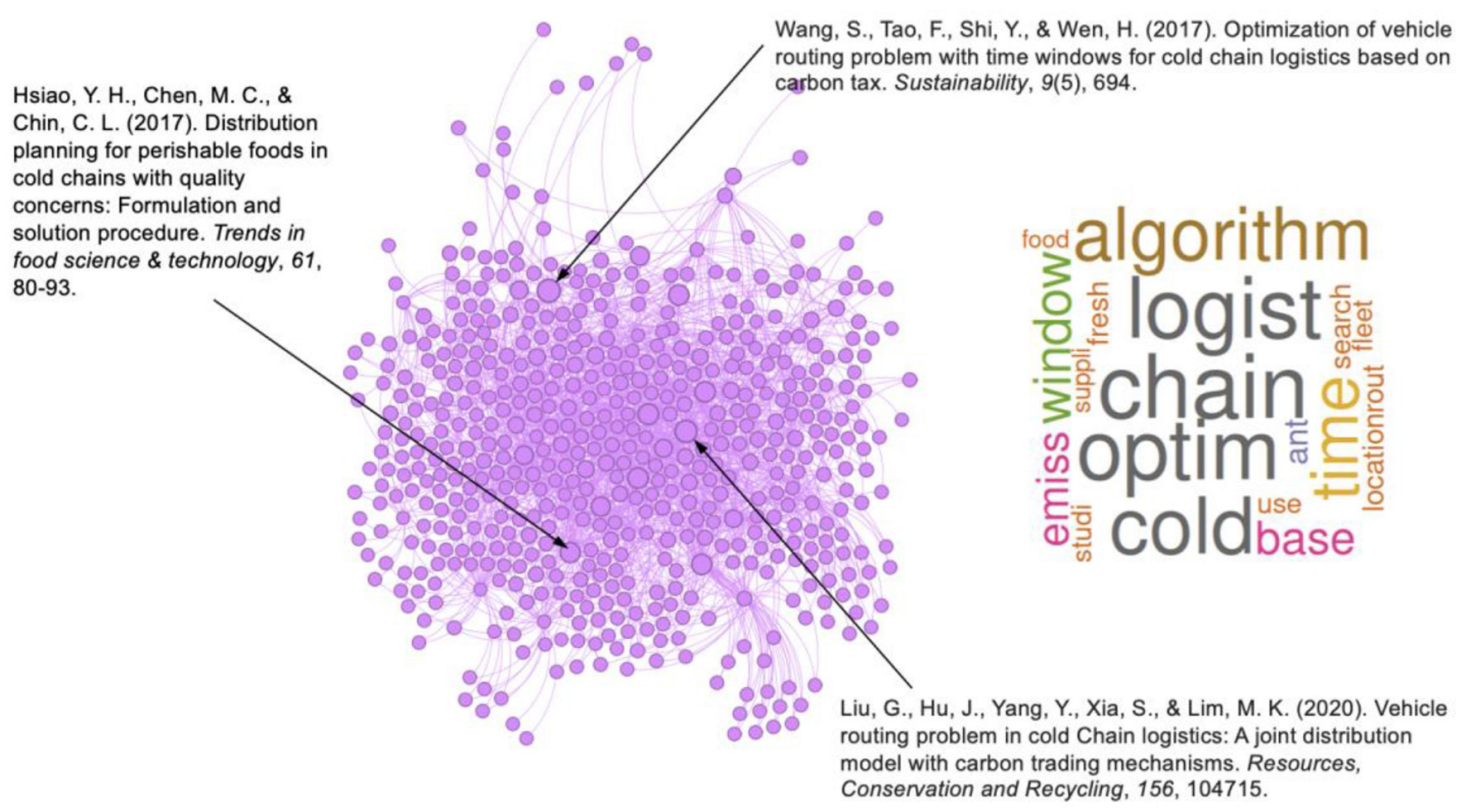
Perspective 1. Algorithmic optimization and mathematical modeling of the RVRP.

The first identified line of research focuses on mathematical modeling and the development of advanced algorithms to solve the refrigerated vehicle routing problem (RVRP). This perspective addresses the problem from a structural, quantitative perspective, formulating extensions of the Vehicle Routing Problem (VRP) that incorporate cold chain constraints, such as strict time windows, thermal control, product deterioration, carbon emissions, and multiple optimization objectives (Figure 5). Studies included in this line prioritize the design of Mixed-Integer Linear Programming (MILP) models, stochastic formulations, multi-objective schemes, and hybrid metaheuristics, with a strong emphasis on computational efficiency and operational realism. Furthermore, a progressive evolution toward integrating sustainability criteria, robustness to uncertainty, and intelligent approaches based on evolutionary techniques and machine learning is observed.

The literature corresponding to this perspective shows an evolution from the classic formulations of the routing problem, based on the seminal truck dispatch model (Dantzig and Ramser, 1959) toward extensions with more complex temporal and structural constraints (Goeke and Schneider, 2015; Ichoua et al., 2003; Xiao et al., 2019). In the specific context of the RVRP, the models explicitly incorporate variables associated with sustainability and the cold chain, including carbon taxes and emissions (Guo et al., 2022; Liu C. et al., 2020; Wang et al., 2017; Zhang L. -Y. et al., 2019), as well as multi-objective approaches that integrate environmental performance and logistics efficiency (Li et al., 2019; Wang et al., 2019; Wang and Wen, 2020). In parallel, there is a consolidation of exact models and MILP formulations for complex variants of the VRP (Franceschetti et al., 2017; Leng et al., 2020), accompanied by a transition toward advanced metaheuristics and hybrid algorithms that seek to solve larger-scale instances (Kim et al., 2016; Liu C. et al., 2020; Song et al., 2020; Zhang H. et al., 2019). In addition, some papers introduce time-dependent dynamics and complex network considerations (Chen et al., 2021; Erdogan and Miller-Hooks, 2012; Xiao and Konak, 2016), while others incorporate computational intelligence and adaptive approaches in multi-objective environments (Liu et al., 2018; Wang and Lu, 2019; Zhao et al., 2020).

Taken together, this corpus reflects a progressive maturation of the RVRP, characterized by the shift from deterministic single-objective models to robust, sustainable, and computationally sophisticated schemes, in which optimization ceases to focus exclusively on cost to integrate environmental impact, operational complexity, and algorithmic scalability.

#### 3.3.2 Perspective 2. Logistics of perishable products and operational management in the cold chain

**Figure 6:**
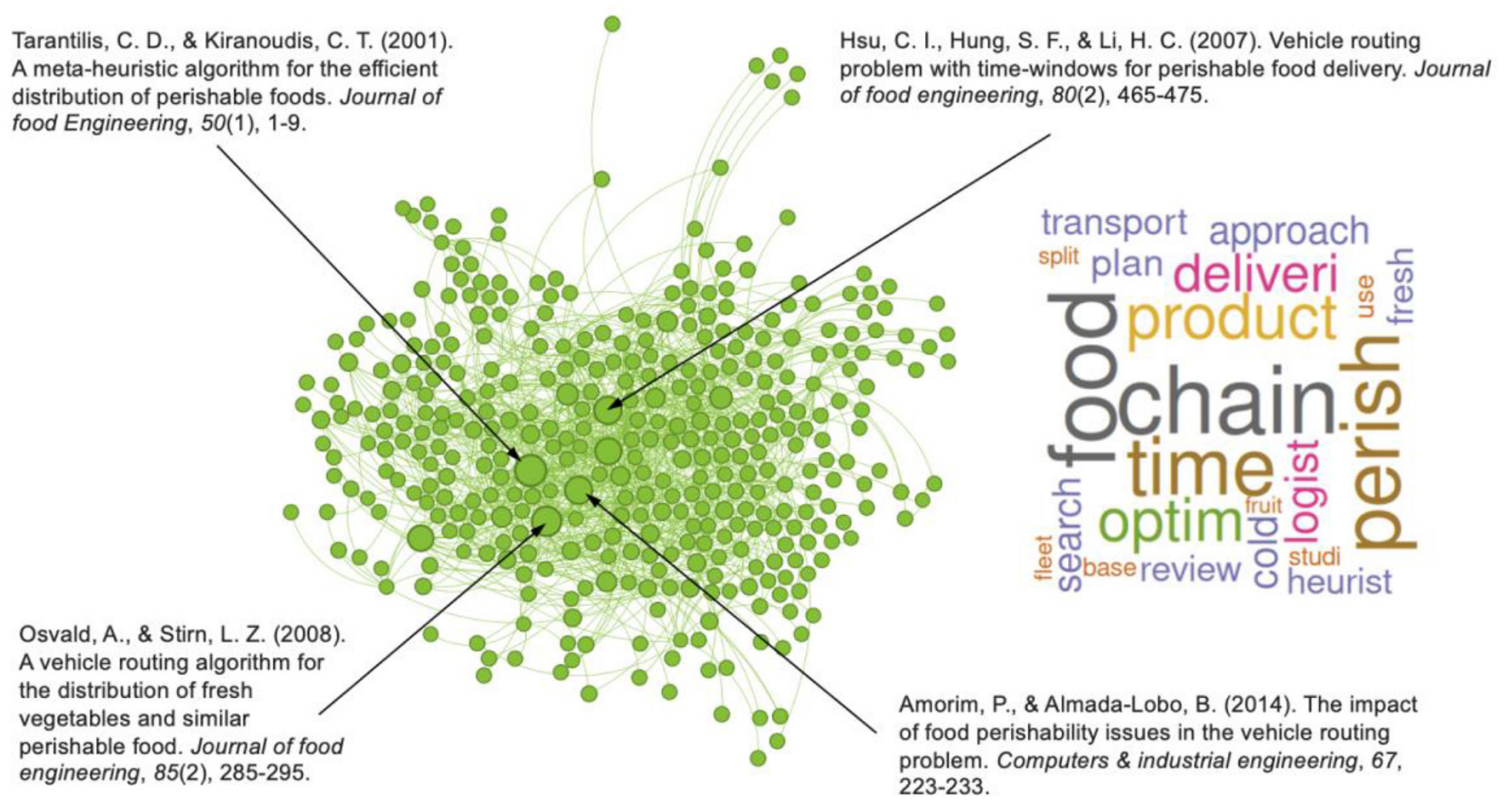
Perspective 2. Logistics of perishable products and operational management in the cold chain.

The second line of research focuses on modeling and optimizing logistics systems for perishable products, with an emphasis on quality degradation, temperature control, and operational performance in the cold chain (Figure 6). Unlike Perspective 1, which centers on algorithmic sophistication, this line prioritizes representing the product's physical and biological behavior (deterioration, shelf life, temperature, quality loss) and integrating it into distribution and planning models (Ahumada and Villalobos, 2011b). Studies combine operations research techniques with time-dependent deterioration models, inventory approaches for products with limited shelf life, and logistics strategies that minimize waste and losses. This line has strong applied connections, particularly in fresh food, the agribusiness sector, and refrigerated logistics, where the objective is not only to minimize costs but also to preserve quality and food safety.

The literature from this perspective shows an early integration between perishable product deterioration modeling and logistics decisions, particularly in studies on quality and shelf life in food products (Tarantilis and Kiranoudis, 2002, 2001; Zhang et al., 2003), as well as applied research in food engineering that incorporates thermal phenomena and heat transfer in cold chain processes (Hsu et al., 2007; James et al., 2006; Osvald and Stirn, 2008; Song and Ko, 2016). Subsequently, these physical and quality approaches are integrated with logistics and production-distribution planning models (Ahumada and Villalobos, 2011a; Govindan et al., 2014; Rong et al., 2011), incorporating shelf-life constraints and inventory policies for time-sensitive products. In parallel, routing and allocation models are developed with specific operational considerations (Amorim and Almada-Lobo, 2014; Chen et al., 2009; Eksioglu et al., 2009; Ma et al., 2017; Pisinger and Ropke, 2007), where deterioration directly influences the objective function. Likewise, methodological contributions are observed that integrate optimization and control under uncertainty and variability in demand or thermal conditions (Ahumada and Villalobos, 2011a; Brito et al., 2012; Savelsbergh, 1985), complemented by applications in logistics management and supply chain (Hsiao et al., 2018; Wang et al., 2018). Finally, some studies apply metaheuristic techniques and hybrid models in perishable goods contexts (Faulin, 2003; Vansteenwegen et al., 2011), consolidating a line of research in which optimization is strongly conditioned by the dynamics of product quality and deterioration. Taken together, this perspective demonstrates a transition from physical-experimental studies of deterioration to integrated logistical-operational models, where quality preservation and waste reduction become central variables in the design of routes and refrigerated distribution systems.

#### 3.3.3 Perspective 3. Inventory-routing integration and strategic logistics coordination models

**Figure 7:**
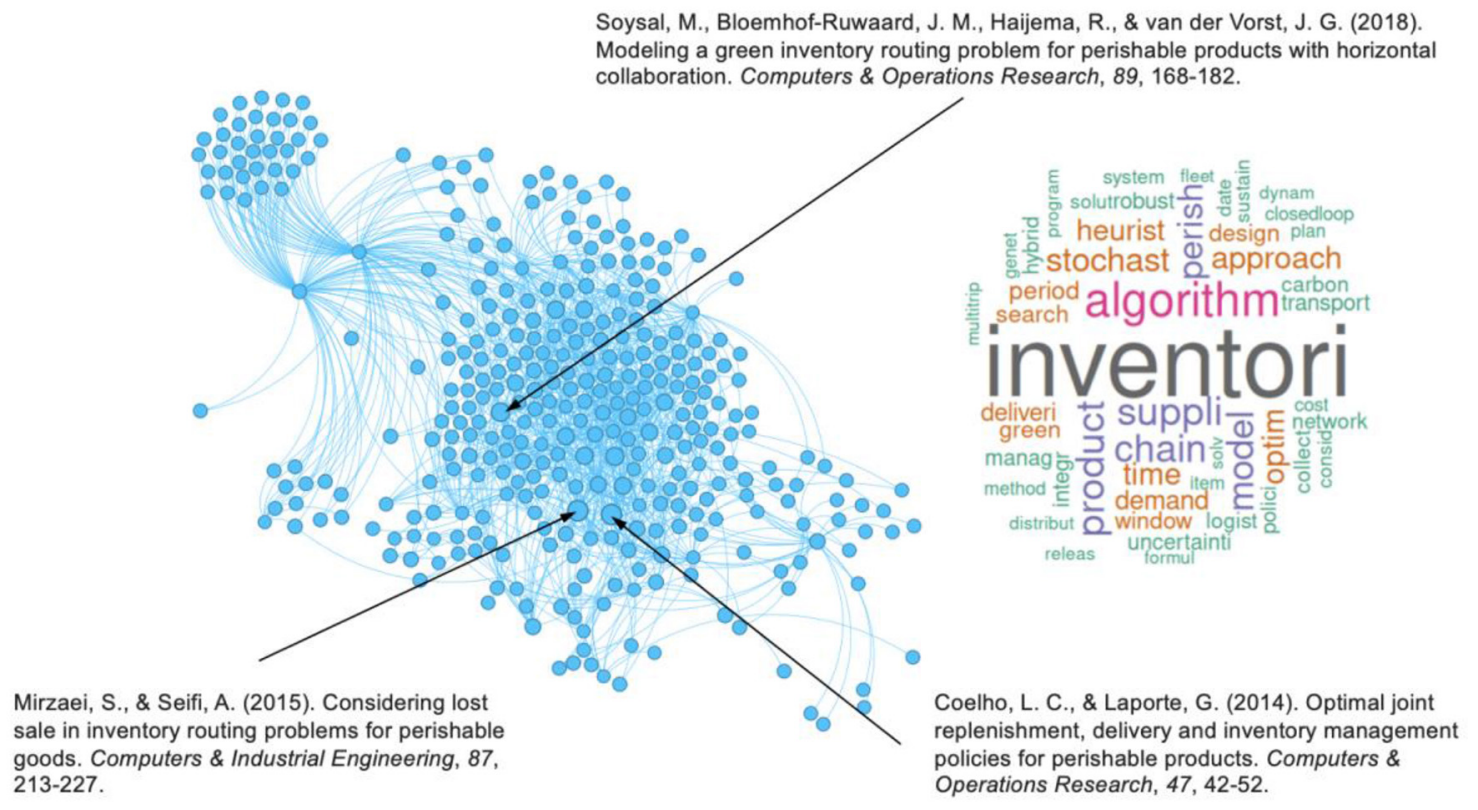
Perspective 3. Inventory-routing integration and strategic logistics coordination models.

The third line of research integrates routing decisions with inventory planning, production, and network design, extending the classic routing problem to coordinated schemes such as the Inventory Routing Problem (IRP) and multi-level strategic models (Figure 7). Unlike the previous perspectives, focused on algorithmic optimization (P1) and spoilage dynamics in perishables (P2). This line addresses the IRP from a systemic perspective, where transportation decisions are not optimized in isolation, but rather in interaction with inventory policies, multi-period horizons, and network structures. Large-scale MILP formulations, multi-period dynamic models, integration with production planning, and supply chain coordination approaches predominate. This perspective reflects a shift toward broader strategic decisions, where the overall system efficiency takes precedence over local route optimization.

The literature grouped in this perspective shows a consolidation of the integrated inventory-routing approach, with early foundations in coordinated transport and planning models (Bell et al., 1983; Beltrami and Bodin, 1974; Federgruen et al., 1986), which later evolved toward more structured formulations of the Inventory Routing Problem (Raa and Aghezzaf, 2009; Soysal et al., 2015; Yu and Nagurney, 2013). In this line, replacement and distribution decisions are modeled jointly, incorporating multi-period horizons and complex operational constraints (Coelho et al., 2012; Coelho and Laporte, 2013b, 2014; Soysal et al., 2018), as well as variants with uncertainty and robustness (Jia et al., 2014; Le et al., 2013). Likewise, contributions are observed from the perspective of network design and strategic coordination in supply chains (Archetti et al., 2007; Cheng et al., 2017; Coelho et al., 2014; Nie and Li, 2013), where transport is linked to long-term structural decisions. In the methodological field, large-scale exact models and mathematical decomposition predominate (Adulyasak et al., 2014; Lenstra and Kan, 1981), complemented by hybrid and heuristic approaches for complex industrial instances (Azadeh et al., 2017; Hiassat et al., 2017; Mirzaei and Seifi, 2015; Shaabani and Kamalabadi, 2016). Furthermore, some studies broaden the scope to include multi-objective coordination and systemic efficiency in integrated environments (Bektaş et al., 2015; Coelho and Laporte, 2013b; Qiu et al., 2019). Taken together, this corpus reveals a maturation of the field toward strategic approaches to logistics coordination, where RVRP is no longer analyzed as an isolated routing problem but becomes a component within integrated supply chain decision systems.

#### 3.3.4 Perspective 4. Dynamic routing, intelligent systems, and multi-objective optimization

**Figure 8:**
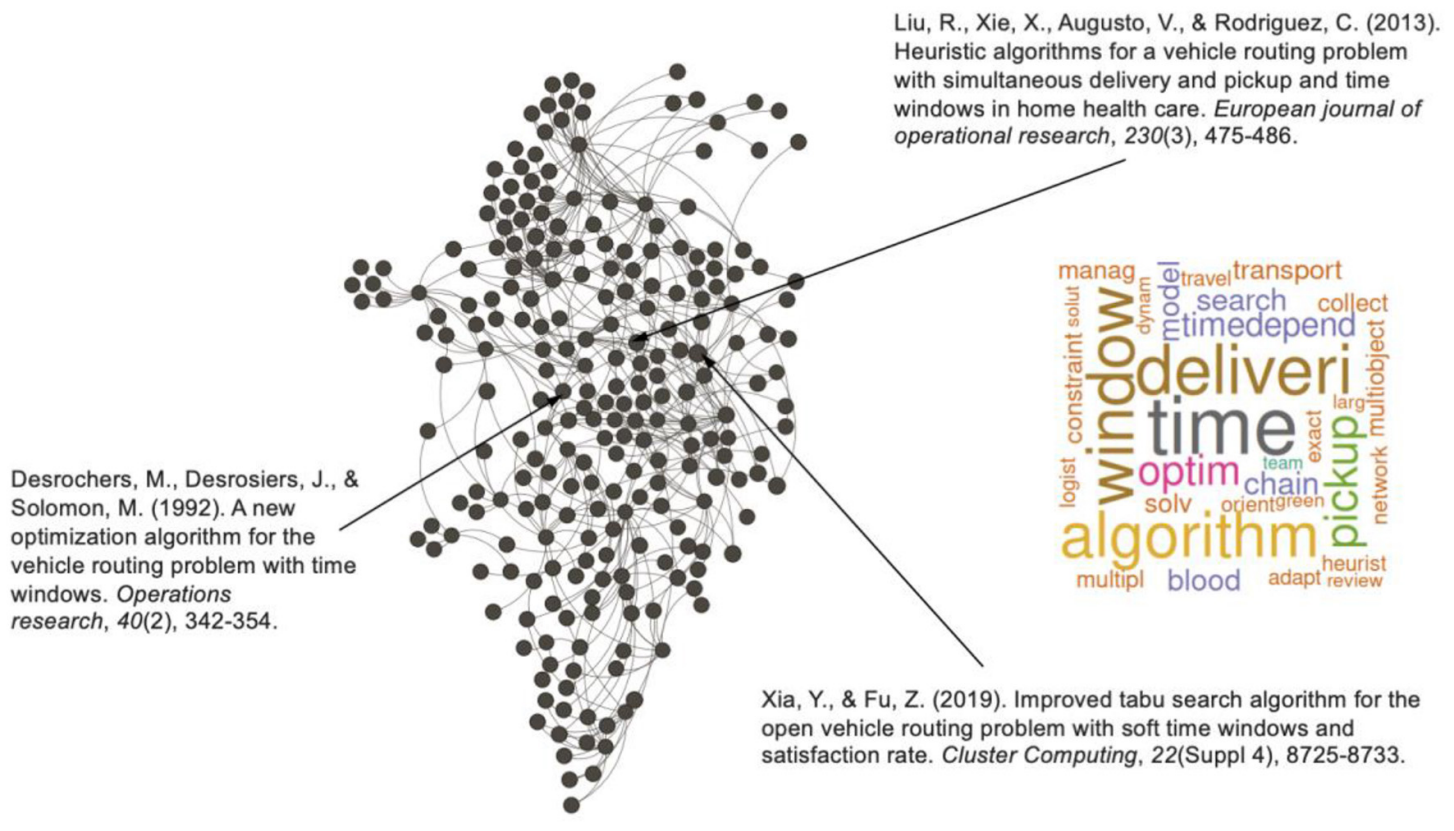
Perspective 4. Dynamic routing, intelligent systems, and multi-objective optimization.

The fourth line of research is characterized by the development of dynamic, time-dependent, and multi-objective variants of the routing problem, integrating smart technologies, urban sustainability, and complex systems (Figure 8). Unlike Perspective 1 (structural algorithmic approach), Perspective 2 (perishables and spoilage), and Perspective 3 (inventory-routing integration), this line focuses on the adaptability of the logistics system to dynamic environments, time-dependent traffic, real-time planning, and multiple objectives that include emissions, resilience, energy efficiency, and urban sustainability. Strong convergence is observed among intelligent transportation systems (ITS), sustainable urban logistics, and hybrid computational models, where routing is integrated with digital technologies and advanced analytics.

The corpus corresponding to this perspective shows an evolution toward dynamic and time-dependent models, particularly in studies on networks with congestion and temporal variability (Alvarez and Munari, 2017; Figliozzi, 2010; Liu et al., 2013), later expanded toward time-dependent and adaptive approaches in urban transport and logistics (Gunpinar and Centeno, 2016; Guo et al., 2023; Hoogeboom et al., 2021; Ulmer et al., 2021). Likewise, there is a consolidation of multi-objective models that integrate environmental criteria and energy sustainability (Erdem, 2022; Moazzeni et al., 2022; Mohammadi et al., 2023), where reductions in emissions and improvements in energy efficiency are explicitly incorporated into the objective function. In the methodological field, hybrid and advanced metaheuristic approaches predominate, applied to large-scale dynamic problems (Luo et al., 2023; Ma et al., 2023; Mobasher et al., 2015), as well as applications in specialized contexts such as hospital logistics and health (Cissé et al., 2017) and intelligent distributed systems (Akbarpour et al., 2021; Xia and Fu, 2019). Fundamental theoretical contributions in optimization and multi-objective modeling (Desrochers et al., 1992; Koch et al., 2018; Parragh et al., 2008) are also identified, providing formal support for recent developments. Taken together, this perspective reflects the transition from RVRP to intelligent, resilient, and sustainable logistics systems, where optimization responds not only to operational constraints but also to highly complex urban, energy, and environmental dynamics.

## 4 Discussion

### 4.1 Cross-perspective synthesis

The structural analysis of the co-citation network reveals that the evolution of the Refrigerated Vehicle Routing Problem (RVRP) cannot be understood as a linear methodological progression, but rather as the consolidation of four partially overlapping research logics. Although each perspective exhibits internal coherence, the cross-perspective synthesis highlights both complementarities and persistent fragmentation within the field.

First, Perspective 1, algorithmic optimization and mathematical modeling, provides the formal backbone of the domain. It advances increasingly sophisticated formulations that incorporate sustainability constraints, stochastic elements, and hybrid metaheuristics. However, when examined alongside Perspective 2, perishable logistics and cold chain operational modeling, a structural asymmetry becomes evident: while optimization models grow algorithmically complex, they often rely on simplified representations of product behavior and thermal dynamics. Conversely, studies grounded in food engineering and deterioration modeling rarely achieve full integration into large-scale routing formulations. This methodological separation suggests that algorithmic refinement has progressed faster than physical realism.

Second, the integration logic represented in Perspective 3, inventory–routing and systemic coordination, partially bridges operational and strategic dimensions. By embedding routing decisions within multi-period planning and supply chain coordination frameworks, this perspective broadens the decision horizon. Nevertheless, the co-citation structure indicates that inventory–routing integration remains more strongly connected to classical operations research than to cold chain-specific modeling. This reinforces the idea that perishability and temperature dynamics are still treated as contextual constraints rather than endogenous structural drivers of the system.

Third, Perspective 4, dynamic, intelligent, and multi-objective routing, introduces adaptability, real-time decision-making, and sustainability transitions. Structurally, this cluster shows greater connectivity to recent work on energy efficiency and emissions. However, its integration with the biological and thermodynamic aspects of cold logistics remains limited. In other words, digital sophistication and environmental optimization evolve in parallel to, rather than fully intertwined with, product-level deterioration processes.

Taken together, the four perspectives reveal a field that is intellectually mature in algorithmic and structural terms but still compartmentalized in its treatment of physical, environmental, and operational realities. The co-citation network shows strong internal density within each perspective but comparatively weaker cross-cluster bridging, suggesting that interdisciplinary integration remains incomplete. The RVRP has thus transitioned from a cost-minimization problem to a sustainability-aware, dynamically adaptive framework; however, the synthesis indicates that true systemic integration, in which thermal variability, deterioration, routing, energy consumption, and supply chain coordination are modeled as mutually dependent processes, has yet to be fully achieved.

This cross-perspective reading, therefore, positions the next stage of research not as further incremental algorithmic refinement, but as deeper conceptual integration across modeling traditions.

### 4.2 Research gaps and future research directions

The scientometric analysis of the four identified research streams reveals significant methodological sophistication across the RVRP literature. However, despite notable advances in algorithmic development, deterioration modeling, inventory-routing integration, and dynamic intelligent systems, several structural gaps remain insufficiently addressed.

#### 4.2.1 Gaps in Perspective 1: algorithmic optimization and mathematical modeling

Although the algorithmic stream has evolved from foundational routing formulations (Dantzig and Ramser, 1959) toward increasingly complex constrained and multi-objective models (Goeke and Schneider, 2015; Xiao et al., 2019), its development remains predominantly optimization centric. Sustainability extensions incorporating carbon taxation and emission metrics (Guo et al., 2022; Wang and Wen, 2020; Zhang H. et al., 2019) largely embed environmental considerations as additional objective terms rather than reconfiguring supply chain structures or decision paradigms.

Furthermore, while advanced metaheuristics and hybrid intelligent methods (Kim et al., 2016; Liu C. et al., 2020; Wang and Lu, 2019) significantly enhance computational scalability, empirical validation in real industrial settings remains limited. Many studies rely on synthetic benchmarks, leaving open questions regarding implementation feasibility, organizational adoption, and long-term performance under operational variability.

Future research should therefore explore:

- Empirical validation through longitudinal industrial case studies.
- Integration of regulatory, behavioral, and governance dimensions.
- Structural redesign approaches beyond objective-function expansion.

#### 4.2.2 Gaps in Perspective 2: perishable logistics and cold chain operations

The second stream successfully integrates product deterioration and thermal dynamics into logistics models (Hsu et al., 2007; James et al., 2006; Tarantilis and Kiranoudis, 2001). However, deterioration processes are frequently modeled using deterministic or simplified decay functions. Even when uncertainty is incorporated (Ahumada and Villalobos, 2011a; Brito et al., 2012), biological variability and real-time environmental fluctuations remain partially abstracted.

Moreover, a simplification in structural modeling persists across this stream: temperature is commonly assumed to remain constant along each transportation arc. Even in routing models that explicitly consider spoilage (Chen et al., 2009; Ma et al., 2017), ambient temperature is typically treated as a fixed parameter rather than a spatially and temporally variable condition. This assumption neglects environmental heterogeneity, including altitude differences between nodes and variations in geographic climate, which may significantly influence refrigeration load, energy consumption, and deterioration rates. Consequently, current models may underestimate the operational complexity of real cold chain systems.

Future research should therefore advance toward dynamic, arc-dependent thermal modeling frameworks that account for real environmental data and altitude-related gradients, enabling a more realistic coupling between deterioration dynamics, energy use, and routing decisions.

Future research directions include:

- Real-time data integration from Internet of Things (IoT)-based temperature monitoring.
- Stochastic biological degradation models linked dynamically to routing decisions.
- Explicit incorporation of food safety regulation and traceability mechanisms into optimization frameworks.

#### 4.2.3 Gaps in Perspective 3: inventory–routing integration and strategic coordination

The third stream expands the analytical scope of routing by integrating inventory and network design decisions (Coelho et al., 2014; Raa and Aghezzaf, 2009; Soysal et al., 2018). While this systemic approach enhances theoretical completeness, increasing dimensionality often yields highly abstract models with limited empirical evidence.

Robust and uncertainty-based variants (Jia et al., 2014; Le et al., 2013) improve resilience modeling, yet uncertainty is frequently treated parametrically rather than adaptively. Moreover, coordination models (Coelho and Laporte, 2013a; Qiu et al., 2019) rarely incorporate behavioral dynamics among supply chain actors.

Future research should address:

- Adaptive learning-based IRP models under real-time demand updates.
- Agent-based or game-theoretical extensions capturing decentralized coordination.
- Implementation studies evaluating trade-offs between model optimality and managerial interpretability.

#### 4.2.4 Gaps in Perspective 4: dynamic, intelligent, and multi-objective systems

The fourth stream advances dynamic and time-dependent routing formulations (Figliozzi, 2010; Guo et al., 2023; Ulmer et al., 2021) and integrates sustainability metrics (Erdem, 2022; Moazzeni et al., 2022; Mohammadi et al., 2023). Nevertheless, the integration of intelligent systems often remains confined to computational performance improvements rather than institutional transformation. Hybrid metaheuristics and adaptive algorithms (Luo et al., 2023; Ma et al., 2023; Pirabán et al., 2019) enhance solution quality but may reduce model interpretability for practitioners and policymakers. Additionally, while urban sustainability is incorporated mathematically, limited research examines regulatory alignment or socio-economic implications.

Future directions include:

- Explainable optimization and interpretable AI in logistics decision support.
- Integration of routing models with urban policy and energy transition frameworks.
- Digital twin architectures connecting real-time data streams with adaptive routing decisions

### 4.3 Future research agenda

The structural synthesis of the RVRP literature suggests that future research should move beyond incremental algorithmic improvements and toward deeper integration across modeling traditions. The evolution of the field reveals both methodological sophistication and conceptual compartmentalization. Addressing this fragmentation requires coordinated advances along four strategic directions.

First, there is a pressing need to develop thermally dynamic routing models that move beyond the widespread assumption of constant temperature along each arc. Most existing formulations treat temperature as a fixed parameter or as a binary feasibility constraint, neglecting spatial and environmental variability. Future models should incorporate temperature as an endogenous and arc-dependent variable, potentially linked to real-world climatic averages, altitude differences between nodes, vehicle insulation performance, and door-opening events. Integrating thermal exposure functions into routing and energy consumption models would allow deterioration, fuel usage, and emissions to be jointly optimized rather than sequentially constrained.

Second, research should aim to integrate physical product dynamics and strategic logistics decisions at multiple scales. While deterioration models exist in food engineering and inventory planning, they are rarely embedded within large-scale routing or network design formulations. A promising direction is to couple heat transfer models, shelf-life kinetics, and routing decisions within unified multi-period optimization frameworks. Such integration would reposition perishability as a structural determinant of routing topology rather than a *post hoc* constraint.

Third, the development of data-driven, adaptive optimization frameworks remains underdeveloped in the refrigerated routing context. Although intelligent and hybrid metaheuristics have gained prominence, the integration of real-time temperature sensing, IoT-enabled monitoring, and predictive analytics into routing decisions is still limited. Future work could explore digital twins of cold chains, reinforcement learning for adaptive temperature-routing coordination, and stochastic models calibrated with empirical temperature and deterioration data.

Fourth, sustainability modeling should transition from emission accounting to energy-thermodynamic coupling. Current multi-objective approaches often include carbon emissions as an additive objective, yet they seldom model the interactions among refrigeration energy demand, vehicle load, ambient temperature, and routing time. Integrating thermodynamic energy models with routing optimization would enable more realistic trade-off analysis between service level, environmental impact, and product quality preservation.

Finally, future studies should prioritize empirical validation and cross-disciplinary collaboration. Much of the RVRP literature remains computational and simulation-based. Field-calibrated models, industry case studies, and experimental validation of thermal-routing interactions would strengthen both theoretical robustness and managerial relevance. Cross-fertilization between operations research, thermal engineering, food science, and transportation systems research appears essential for the next phase of the field's maturation.

In sum, the future trajectory of the RVRP should shift from algorithmic refinement to systemic integration, in which routing, deterioration, energy consumption, and environmental conditions are modeled as interdependent processes within a unified decision framework.

## 5 Conclusions

This study mapped the intellectual evolution of the Refrigerated Vehicle Routing Problem (RVRP) through a co-citation network analysis of 469 articles indexed in the Web of Science Core Collection. By integrating modularity-based community detection, PageRank centrality analysis, and keyword frequency examination, the research identified four dominant perspectives shaping the field: (1) algorithmic optimization and mathematical modeling, (2) perishable logistics and cold chain operations, (3) inventory–routing integration and strategic coordination, and (4) dynamic, intelligent, and multi-objective routing systems.

The structural properties of the co-citation network reveal a cohesive yet moderately modular intellectual architecture. Although nine communities were detected algorithmically, four dominant clusters concentrate the most influential and structurally central contributions. This finding indicates a mature research domain characterized by strong intra-perspective consolidation but comparatively limited cross-perspective integration.

The cross-perspective synthesis demonstrates that the RVRP has evolved beyond traditional cost-minimization paradigms toward sustainability-aware and computationally sophisticated formulations. However, the analysis also uncovers persistent fragmentation. Algorithmic advancements frequently rely on simplified thermal and deterioration assumptions, while physically grounded cold chain studies rarely integrate fully with large-scale routing optimization. Similarly, inventory–routing and dynamic intelligent systems approaches expand decision horizons but often treat thermodynamic variability as an exogenous constraint rather than an endogenous structural driver.

One of the most critical structural gaps identified across perspectives is the widespread assumption of constant temperature along routing arcs. Despite advances in energy-efficient routing and carbon-aware optimization, few studies incorporate spatially and temporally variable thermal exposure, altitude gradients, or real climatic heterogeneity into routing formulations. This limitation suggests that the thermodynamic dimension of refrigerated logistics remains only partially integrated into optimization frameworks.

The findings suggest that the next stage in the evolution of the RVRP should move from incremental algorithmic refinement toward systemic integration. Future research should focus on coupling routing decisions with dynamic thermal modeling, deterioration kinetics, energy consumption, and environmental variability within unified multi-scale decision systems. Such integration would enable more realistic trade-off analyses between service level, sustainability, and product quality preservation.

From a methodological standpoint, this study contributes a reproducible scientometric framework for mapping and synthesizing the intellectual structure of specialized VRP variants. From a theoretical perspective, it advances the conceptual consolidation of the RVRP domain by revealing structural complementarities and gaps across modeling traditions. Finally, from a practical perspective, the study highlights the need for empirically validated and physically grounded routing models capable of addressing the increasing complexity of real-world cold chain logistics.

## Data Availability

The original contributions presented in the study are included in the article/supplementary material, further inquiries can be directed to the corresponding author.

## References

[B1] Adulyasak Y. Cordeau J.-F. Jans R. (2014). Formulations and branch-and-cut algorithms for multivehicle production and inventory routing problems. INFORMS J. Comput. 26, 103–120. doi: 10.1287/ijoc.2013.0550

[B2] Ahumada O. Villalobos J. R. (2011a). Operational model for planning the harvest and distribution of perishable agricultural products. Int. J. Prod. Econ. 133, 677–687. doi: 10.1016/j.ijpe.2011.05.015

[B3] Ahumada O. Villalobos J. R. (2011b). A tactical model for planning the production and distribution of fresh produce. Ann. Oper. Res. 190, 339–358. doi: 10.1007/s10479-009-0614-4

[B4] Akbarpour N. Salehi-Amiri A. Hajiaghaei-Keshteli M. Oliva D. (2021). An innovative waste management system in a smart city under stochastic optimization using vehicle routing problem. Soft. Comput. 25, 6707–6727. doi: 10.1007/s00500-021-05669-6

[B5] Alvarez A. Munari P. (2017). An exact hybrid method for the vehicle routing problem with time windows and multiple deliverymen. Comput. Oper. Res. 83, 1–12. doi: 10.1016/j.cor.2017.02.001

[B6] Amorim P. Almada-Lobo B. (2014). The impact of food perishability issues in the vehicle routing problem. Comput. Ind. Eng. 67, 223–233. doi: 10.1016/j.cie.2013.11.006

[B7] Archetti C. Bertazzi L. Laporte G. Speranza M. G. (2007). A branch-and-cut algorithm for a vendor-managed inventory-routing problem. Transp. Sci. 41, 382–391. doi: 10.1287/trsc.1060.0188

[B8] Aria M. Cuccurullo C. (2017). Bibliometrix : an R-tool for comprehensive science mapping analysis. J. Infor. 11, 959–975. doi: 10.1016/j.joi.2017.08.007

[B9] Azadeh A. Elahi S. Farahani M. H. Nasirian B. (2017). A genetic algorithm-Taguchi based approach to inventory routing problem of a single perishable product with transshipment. Comput. Ind. Eng. 104, 124–133. doi: 10.1016/j.cie.2016.12.019

[B10] Barrera Rodríguez A. M. Duque Oliva E. J. Vieira Salazar J. A. (2023). Actor engagement: origin, evolution and trends. JBIM 38, 1479–1497. doi: 10.1108/JBIM-11-2021-0512

[B11] Bektaş T. Laporte G. Vigo D. (2015). Integrated vehicle routing problems. Comput. Oper. Res. 55:126. doi: 10.1016/j.cor.2014.08.008

[B12] Bell W. J. Dalberto L. M. Fisher M. L. Greenfield A. J. Jaikumar R. Kedia P. . (1983). Improving the distribution of industrial gases with an on-line computerized routing and scheduling optimizer. Interfaces 13, 4–23. doi: 10.1287/inte.13.6.4

[B13] Beltrami E. J. Bodin L. D. (1974). Networks and vehicle routing for municipal waste collection. Networks 4, 65–94. doi: 10.1002/net.3230040106

[B14] Brito J. Martinez F. J. Moreno J. A. Verdegay J. L. (2012). Fuzzy optimization for distribution of frozen food with imprecise times. Fuzzy Optim. Decis. Mak. 11, 337–349. doi: 10.1007/s10700-012-9131-z

[B15] Chen H.-K. Hsueh C.-F. Chang M.-S. (2009). Production scheduling and vehicle routing with time windows for perishable food products. Comput. Oper. Res. 36, 2311–2319. doi: 10.1016/j.cor.2008.09.010

[B16] Chen J. Liao W. Yu C. (2021). Route optimization for cold chain logistics of front warehouses based on traffic congestion and carbon emission. Comput. Ind. Eng. 161:107663. doi: 10.1016/j.cie.2021.107663

[B17] Cheng C. Yang P. Qi M. Rousseau L.-M. (2017). Modeling a green inventory routing problem with a heterogeneous fleet. Transp. Res. E: Logist. Transp. Rev. 97, 97–112. doi: 10.1016/j.tre.2016.11.001

[B18] Cissé M. Yalçindag S. Kergosien Y. Sahin E. Lenté C. Matta A. (2017). OR problems related to home health care: a review of relevant routing and scheduling problems. Oper. Res. Health Care 13–14, 1–22. doi: 10.1016/j.orhc.2017.06.001

[B19] Coelho L. C. Cordeau J.-F. Laporte G. (2012). The inventory-routing problem with transshipment. Comput. Oper. Res. 39, 2537–2548. doi: 10.1016/j.cor.2011.12.020

[B20] Coelho L. C. Cordeau J.-F. Laporte G. (2014). Thirty years of inventory routing. Transp. Sci. 48, 1–19. doi: 10.1287/trsc.2013.0472

[B21] Coelho L. C. Laporte G. (2013a). The exact solution of several classes of inventory-routing problems. Comput. Oper. Res. 40, 558–565. doi: 10.1016/j.cor.2012.08.012

[B22] Coelho L. C. Laporte G. (2013b). A branch-and-cut algorithm for the multi-product multi-vehicle inventory-routing problem. Int. J. Prod. Res. 51, 7156–7169. doi: 10.1080/00207543.2012.757668

[B23] Coelho L. C. Laporte G. (2014). Optimal joint replenishment, delivery and inventory management policies for perishable products. Comput. Oper. Res. 47, 42–52. doi: 10.1016/j.cor.2014.01.013

[B24] Dantzig G. B. Ramser J. H. (1959). The truck dispatching problem. Manag. Sci. 6, 80–91. doi: 10.1287/mnsc.6.1.80

[B25] Desrochers M. Desrosiers J. Solomon M. (1992). A new optimization algorithm for the vehicle routing problem with time windows. Oper. Res. 40, 342–354. doi: 10.1287/opre.40.2.342

[B26] Donthu N. Kumar S. Mukherjee D. Pandey N. Lim W. M. (2021a). How to conduct a bibliometric analysis: an overview and guidelines. J. Bus. Res. 133, 285–296. doi: 10.1016/j.jbusres.2021.04.070

[B27] Donthu N. Kumar S. Pandey N. Gupta P. (2021b). Forty years of the international journal of information management: a bibliometric analysis. Int. J. Inf. Manag. 57:102307. doi: 10.1016/j.ijinfomgt.2020.102307

[B28] Eksioglu B. Vural A. V. Reisman A. (2009). The vehicle routing problem: a taxonomic review. Comput. Ind. Eng. 57, 1472–1483. doi: 10.1016/j.cie.2009.05.009

[B29] Erdem M. (2022). Optimisation of sustainable urban recycling waste collection and routing with heterogeneous electric vehicles. Sustain. Cities Soc. 80:103785. doi: 10.1016/j.scs.2022.103785

[B30] Erdogan S. Miller-Hooks E. (2012). A green vehicle routing problem. Transp. Res. E: Logist. Transp. Rev. 48, 100–114. doi: 10.1016/j.tre.2011.08.001

[B31] Escobar J. W. Duque J. L. R. García-Cáceres R. (2022). A granular tabu search for the refrigerated vehicle routing problem with homogeneous fleet. Int. J. Ind. Eng. Comput. 13, 135–150. doi: 10.5267/j.ijiec.2021.6.001

[B32] Faulin J. (2003). Applying MIXALG procedure in a routing problem to optimize food product delivery. Omega 31, 387–395. doi: 10.1016/S0305-0483(03)00079-3

[B33] Federgruen A. Prastacos G. Zipkin P. H. (1986). An allocation and distribution model for perishable products. Oper. Res. 34, 75–82. doi: 10.1287/opre.34.1.75

[B34] Figliozzi M. A. (2010). An iterative route construction and improvement algorithm for the vehicle routing problem with soft time windows. Transp. Res. C: Emerg. Technol. 18, 668–679. doi: 10.1016/j.trc.2009.08.005

[B35] Franceschetti A. Demir E. Honhon D. Van Woensel T. Laporte G. Stobbe M. (2017). A metaheuristic for the time-dependent pollution-routing problem. Eur. J. Oper. Res. 259, 972–991. doi: 10.1016/j.ejor.2016.11.026

[B36] Goeke D. Schneider M. (2015). Routing a mixed fleet of electric and conventional vehicles. Eur. J. Oper. Res. 245, 81–99. doi: 10.1016/j.ejor.2015.01.049

[B37] Golden B. Raghavan S. Wasil E. (Eds.). (2008). The Vehicle Routing Problem: Latest Advances and New Challenges, Operations Research/Computer Science Interfaces. Boston, MA: Springer. doi: 10.1007/978-0-387-77778-8

[B38] Govindan K. Jafarian A. Khodaverdi R. Devika K. (2014). Two-echelon multiple-vehicle location–routing problem with time windows for optimization of sustainable supply chain network of perishable food. Int. J. Prod. Econ. 152, 9–28. doi: 10.1016/j.ijpe.2013.12.028

[B39] Gunpinar S. Centeno G. (2016). An integer programming approach to the bloodmobile routing problem. Transp. Res. E: Logist. Transp. Rev. 86, 94–115. doi: 10.1016/j.tre.2015.12.005

[B40] Guo F. Wei Q. Wang M. Guo Z. Wallace S. W. (2023). Deep attention models with dimension-reduction and gate mechanisms for solving practical time-dependent vehicle routing problems. Transp. Res. E: Logist. Transp. Rev. 173:103095. doi: 10.1016/j.tre.2023.103095

[B41] Guo X. Zhang W. Liu B. (2022). Low-carbon routing for cold-chain logistics considering the time-dependent effects of traffic congestion. Transp. Res. D: Transp. Environ. 113:103502. doi: 10.1016/j.trd.2022.103502

[B42] Hiassat A. Diabat A. Rahwan I. (2017). A genetic algorithm approach for location-inventory-routing problem with perishable products. J. Manuf. Syst. 42, 93–103. doi: 10.1016/j.jmsy.2016.10.004

[B43] Hoogeboom M. Adulyasak Y. Dullaert W. Jaillet P. (2021). The robust vehicle routing problem with time window assignments. Transp. Sci. 55, 395–413. doi: 10.1287/trsc.2020.1013

[B44] Hsiao Y.-H. Chen M.-C. Lu K.-Y. Chin C.-L. (2018). Last-mile distribution planning for fruit-and-vegetable cold chains. IJLM 29, 862–886. doi: 10.1108/IJLM-01-2017-0002

[B45] Hsu C.-I. Hung S.-F. Li H.-C. (2007). Vehicle routing problem with time-windows for perishable food delivery. J. Food Eng. 80, 465–475. doi: 10.1016/j.jfoodeng.2006.05.029

[B46] Ichoua S. Gendreau M. Potvin J.-Y. (2003). Vehicle dispatching with time-dependent travel times. Eur. J. Oper. Res. 144, 379–396. doi: 10.1016/S0377-2217(02)00147-9

[B47] James S. J. James C. Evans J. A. (2006). Modelling of food transportation systems – a review. Int. J. Refrig. 29, 947–957. doi: 10.1016/j.ijrefrig.2006.03.017

[B48] Jia T. Li X. Wang N. Li R. (2014). Integrated inventory routing problem with quality time windows and loading cost for deteriorating items under discrete time. Math. Probl. Eng. 2014:537409. doi: 10.1155/2014/537409

[B49] Kim K. Kim H. Kim S.-K. Jung J.-Y. (2016). i-RM: an intelligent risk management framework for context-aware ubiquitous cold chain logistics. Expert Syst. Appl. 46, 463–473. doi: 10.1016/j.eswa.2015.11.005

[B50] Koch H. Bortfeldt A. Wäscher G. (2018). A hybrid algorithm for the vehicle routing problem with backhauls, time windows and three-dimensional loading constraints. OR Spectr. 40, 1029–1075. doi: 10.1007/s00291-018-0506-6

[B51] Le T. Diabat A. Richard J.-P. Yih Y. (2013). A column generation-based heuristic algorithm for an inventory routing problem with perishable goods. Optim. Lett. 7, 1481–1502. doi: 10.1007/s11590-012-0540-2

[B52] Leng L. Zhang J. Zhang C. Zhao Y. Wang W. Li G. (2020). Decomposition-based hyperheuristic approaches for the bi-objective cold chain considering environmental effects. Comput. Oper. Res. 123:105043. doi: 10.1016/j.cor.2020.105043

[B53] Lenstra J. K. Kan A. H. G. R. (1981). Complexity of vehicle routing and scheduling problems. Networks 11, 221–227. doi: 10.1002/net.3230110211

[B54] Li Y. Soleimani H. Zohal M. (2019). An improved ant colony optimization algorithm for the multi-depot green vehicle routing problem with multiple objectives. J. Clean. Prod. 227, 1161–1172. doi: 10.1016/j.jclepro.2019.03.185

[B55] Liu C. Kou G. Zhou X. Peng Y. Sheng H. Alsaadi F. E. (2020). Time-dependent vehicle routing problem with time windows of city logistics with a congestion avoidance approach. Knowl.-Based Syst. 188:104813. doi: 10.1016/j.knosys.2019.06.021

[B56] Liu G. Hu J. Yang Y. Xia S. Lim M. K. (2020). Vehicle routing problem in cold Chain logistics: a joint distribution model with carbon trading mechanisms. Resour. Conserv. Recycl. 156:104715. doi: 10.1016/j.resconrec.2020.104715

[B57] Liu H. Pretorius L. Jiang D. (2018). Optimization of cold chain logistics distribution network terminal. J. Wirel. Commun. Netw. 2018:158. doi: 10.1186/s13638-018-1168-4

[B58] Liu R. Xie X. Augusto V. Rodriguez C. (2013). Heuristic algorithms for a vehicle routing problem with simultaneous delivery and pickup and time windows in home health care. Eur. J. Oper. Res. 230, 475–486. doi: 10.1016/j.ejor.2013.04.044

[B59] Luo H. Dridi M. Grunder O. (2023). A branch-price-and-cut algorithm for a time-dependent green vehicle routing problem with the consideration of traffic congestion. Comput. Ind. Eng. 177:109093. doi: 10.1016/j.cie.2023.109093

[B60] Ma B. Hu D. Wang Y. Sun Q. He L. Chen X. (2023). Time-dependent vehicle routing problem with departure time and speed optimization for shared autonomous electric vehicle service. Appl. Math. Model. 113, 333–357. doi: 10.1016/j.apm.2022.09.020

[B61] Ma Z.-J. Wu Y. Dai Y. (2017). A combined order selection and time-dependent vehicle routing problem with time widows for perishable product delivery. Comput. Ind. Eng. 114, 101–113. doi: 10.1016/j.cie.2017.10.010

[B62] Meneghetti A. Ceschia S. (2020). Energy-efficient frozen food transports: the refrigerated routing problem. Int. J. Prod. Res. 58, 4164–4181. doi: 10.1080/00207543.2019.1640407

[B63] Mirzaei S. Seifi A. (2015). Considering lost sale in inventory routing problems for perishable goods. Comput. Ind. Eng. 87, 213–227. doi: 10.1016/j.cie.2015.05.010

[B64] Moazzeni S. Tavana M. Mostafayi Darmian S. (2022). A dynamic location-arc routing optimization model for electric waste collection vehicles. J. Clean. Prod. 364:132571. doi: 10.1016/j.jclepro.2022.132571

[B65] Mobasher A. Ekici A. Özener O. Ö. (2015). Coordinating collection and appointment scheduling operations at the blood donation sites. Comput. Ind. Eng. 87, 260–266. doi: 10.1016/j.cie.2015.05.020

[B66] Mohammadi M. Rahmanifar G. Hajiaghaei-Keshteli M. Fusco G. Colombaroni C. Sherafat A. (2023). A dynamic approach for the multi-compartment vehicle routing problem in waste management. Renew. Sustain. Energy Rev. 184:113526. doi: 10.1016/j.rser.2023.113526

[B67] Nie Y. Li Q. (2013). An eco-routing model considering microscopic vehicle operating conditions. Transp. Res. B Methodol. 55, 154–170. doi: 10.1016/j.trb.2013.06.004

[B68] Osvald A. Stirn L. Z. (2008). A vehicle routing algorithm for the distribution of fresh vegetables and similar perishable food. J. Food Eng. 85, 285–295. doi: 10.1016/j.jfoodeng.2007.07.008

[B69] Parragh S. N. Doerner K. F. Hartl R. F. (2008). A survey on pickup and delivery problems: Part I: transportation between customers and depot. J. Betriebswirtschaft 58, 21–51. doi: 10.1007/s11301-008-0033-7

[B70] Pirabán A. Guerrero W. J. Labadie N. (2019). Survey on blood supply chain management: models and methods. Comput. Oper. Res. 112:104756. doi: 10.1016/j.cor.2019.07.014

[B71] Pisinger D. Ropke S. (2007). A general heuristic for vehicle routing problems. Comput. Oper. Res. 34, 2403–2435. doi: 10.1016/j.cor.2005.09.012

[B72] Qiu Y. Qiao J. Pardalos P. M. (2019). Optimal production, replenishment, delivery, routing and inventory management policies for products with perishable inventory. Omega 82, 193–204. doi: 10.1016/j.omega.2018.01.006

[B73] Raa B. Aghezzaf E.-H. (2009). A practical solution approach for the cyclic inventory routing problem. Eur. J. Oper. Res. 192, 429–441. doi: 10.1016/j.ejor.2007.09.032

[B74] Robledo S. Duque P. Aguirre A. M. G. (2023). Word of mouth marketing: a scientometric analysis. J. Scientometr. Res. 11, 436–446. doi: 10.5530/jscires.11.3.47

[B75] Rong A. Akkerman R. Grunow M. (2011). An optimization approach for managing fresh food quality throughout the supply chain. Int. J. Prod. Econ. 131, 421–429. doi: 10.1016/j.ijpe.2009.11.026

[B76] Savelsbergh M. W. P. (1985). Local search in routing problems with time windows. Ann. Oper. Res. 4, 285–305. doi: 10.1007/BF02022044

[B77] Shaabani H. Kamalabadi I. N. (2016). An efficient population-based simulated annealing algorithm for the multi-product multi-retailer perishable inventory routing problem. Comput. Ind. Eng. 99, 189–201. doi: 10.1016/j.cie.2016.07.022

[B78] Small H. (1973). Co-citation in the scientific literature: a new measure of the relationship between two documents. J. Am. Soc. Inf. Sci. 24, 265–269. doi: 10.1002/asi.4630240406

[B79] Song B. D. Ko Y. D. (2016). A vehicle routing problem of both refrigerated- and general-type vehicles for perishable food products delivery. J. Food Eng. 169, 61–71. doi: 10.1016/j.jfoodeng.2015.08.027

[B80] Song M. Li J. Han Y. -qi. Han Y. -yan. Liu L. Sun Q. (2020). Metaheuristics for solving the vehicle routing problem with the time windows and energy consumption in cold chain logistics. Appl. Soft Comput. 95:106561. doi: 10.1016/j.asoc.2020.106561

[B81] Soysal M. Bloemhof-Ruwaard J. M. Haijema R. Van Der Vorst J. G. A. J. (2015). Modeling an inventory routing problem for perishable products with environmental considerations and demand uncertainty. Int. J. Prod. Econ. 164, 118–133. doi: 10.1016/j.ijpe.2015.03.008

[B82] Soysal M. Bloemhof-Ruwaard J. M. Haijema R. Van Der Vorst J. G. A. J. (2018). Modeling a green inventory routing problem for perishable products with horizontal collaboration. Comput. Oper. Res. 89, 168–182. doi: 10.1016/j.cor.2016.02.003

[B83] Tarantilis C. D. Kiranoudis C. T. (2001). A meta-heuristic algorithm for the efficient distribution of perishable foods. J. Food Eng. 50, 1–9. doi: 10.1016/S0260-8774(00)00187-4

[B84] Tarantilis C. D. Kiranoudis C. T. (2002). Distribution of fresh meat. J. Food Eng. 51, 85–91. doi: 10.1016/S0260-8774(01)00040-1

[B85] Toth P. Vigo D. (Eds.). (2014). Vehicle Routing: Problems, Methods, and Applications, (2nd Edn.). Philadelphia, PA: Society for Industrial and Applied Mathematics. doi: 10.1137/1.9781611973594

[B86] Ulmer M. W. Thomas B. W. Campbell A. M. Woyak N. (2021). The restaurant meal delivery problem: dynamic pickup and delivery with deadlines and random ready times. Transp. Sci. 55, 75–100. doi: 10.1287/trsc.2020.1000

[B87] Vansteenwegen P. Souffriau W. Berghe G. V. Oudheusden D. V. (2011). The city trip planner: an expert system for tourists. Expert Syst. Appl. 38, 6540–6546. doi: 10.1016/j.eswa.2010.11.085

[B88] Wang L. Lu J. (2019). A memetic algorithm with competition for the capacitated green vehicle routing problem. IEEE/CAA J. Autom. Sin. 6, 516–526. doi: 10.1109/JAS.2019.1911405

[B89] Wang S. Tao F. Shi Y. Wen H. (2017). Optimization of vehicle routing problem with time windows for cold chain logistics based on carbon tax. Sustainability 9:694. doi: 10.3390/su9050694

[B90] Wang X. P. Wang M. Ruan J. H. Li Y. (2018). Multi-objective optimization for delivering perishable products with mixed time windows. Adv. Prod. Eng. Manag. 13, 321–332. doi: 10.14743/apem2018.3.293

[B91] Wang Y. Assogba K. Fan J. Xu M. Liu Y. Wang H. (2019). Multi-depot green vehicle routing problem with shared transportation resource: integration of time-dependent speed and piecewise penalty cost. J. Clean. Prod. 232, 12–29. doi: 10.1016/j.jclepro.2019.05.344

[B92] Wang Z. Wen P. (2020). Optimization of a low-carbon two-echelon heterogeneous-fleet vehicle routing for cold chain logistics under mixed time window. Sustainability 12:1967. doi: 10.3390/su12051967

[B93] Xia Y. Fu Z. (2019). Improved tabu search algorithm for the open vehicle routing problem with soft time windows and satisfaction rate. Clust. Comput. 22:8725–8733. doi: 10.1007/s10586-018-1957-x

[B94] Xiao Y. Konak A. (2016). The heterogeneous green vehicle routing and scheduling problem with time-varying traffic congestion. Transp. Res. E 88, 146–166. doi: 10.1016/j.tre.2016.01.011

[B95] Xiao Y. Zuo X. Kaku I. Zhou S. Pan X. (2019). Development of energy consumption optimization model for the electric vehicle routing problem with time windows. J. Clean. Prod. 225, 647–663. doi: 10.1016/j.jclepro.2019.03.323

[B96] Yu M. Nagurney A. (2013). Competitive food supply chain networks with application to fresh produce. Eur. J. Oper. Res. 224, 273–282. doi: 10.1016/j.ejor.2012.07.033

[B97] Zhang G. Habenicht W. Ludwig Spieß W. E. (2003). Improving the structure of deep frozen and chilled food chain with tabu search procedure. J. Food Eng. 60, 67–79. doi: 10.1016/S0260-8774(03)00019-0

[B98] Zhang H. Zhang Q. Ma L. Zhang Z. Liu Y. (2019). A hybrid ant colony optimization algorithm for a multi-objective vehicle routing problem with flexible time windows. Inform. Sci. 490, 166–190. doi: 10.1016/j.ins.2019.03.070

[B99] Zhang L. -Y. Tseng M. -L. Wang C. -H. Xiao C. Fei T. (2019). Low-carbon cold chain logistics using ribonucleic acid-ant colony optimization algorithm. J. Clean. Prod. 233, 169–180. doi: 10.1016/j.jclepro.2019.05.306

[B100] Zhao B. Gui H. Li H. Xue J. (2020). Cold chain logistics path optimization via improved multi-objective ant colony algorithm. IEEE Access 8, 142977–142995. doi: 10.1109/ACCESS.2020.3013951

[B101] Zupic I. Cater T. (2015). Bibliometric methods in management and organization. Organ. Res. Methods 18, 429–472. doi: 10.1177/1094428114562629

